# The Neutrophil-to-Lymphocyte Ratio is Associated with the Requirement and the Duration of Invasive Mechanical Ventilation in Acute Respiratory Distress Syndrome Patients: A Retrospective Study

**DOI:** 10.1155/2022/1581038

**Published:** 2022-07-16

**Authors:** Lijuan Yang, Chang Gao, Ying He, Xiaowan Wang, Ling Yang, Shiqi Guo, Jiahao Chen, Siyu He, Yuanxiao Sun, Ye Gao, Qiang Guo

**Affiliations:** ^1^Department of Critical Care Medicine, The First Affiliated Hospital of Soochow University, Suzhou, Jiangsu, China; ^2^Department of Emergency and Critical Care Medicine, Dushu Lake Hospital Affiliated to Soochow University (Suzhou Dushu Lake Hospital), Suzhou, Jiangsu, China; ^3^Medical Center of Soochow University, Suzhou, Jiangsu, China; ^4^Institute of Critical Care Medicine, Soochow University, Suzhou, Jiangsu, China; ^5^Department of Critical Care Medicine, Taicang Affiliated Hospital of Soochow University, Suzhou, Jiangsu, China

## Abstract

**Background:**

Acute respiratory distress syndrome (ARDS) is associated with high in-hospital mortality and most ARDS patients require ventilatory support. Applying appropriate ventilation strategies based on patients' individual situations has a direct impact upon patients' outcome. The neutrophil-to-lymphocyte ratio (NLR) has been shown to predict the early requirement of invasive mechanical ventilation (IMV) in patients with coronavirus disease 2019 (COVID-19). Our study aimed to investigate the relationship between baseline NLR and IMV in ARDS.

**Methods:**

A retrospective study was performed on patients who were diagnosed with ARDS using the Berlin definition and admitted to the First Affiliated Hospital of Soochow University from 2017 to 2022. Clinical data within 24 h after the ARDS diagnosis were collected from the medical record system. Based on the ventilation strategies during hospitalization, patients were divided into three groups and their clinical characteristics were compared. Furthermore, logistic regression analysis was used to screen the independent risk factors for IMV. STROBE checklist was used for this manuscript.

**Results:**

520 ARDS patients were included and the median NLR value in IMV group was significantly higher than that of other groups (*P* < 0.001). NLR was significantly associated with the requirement of IMV in ARDS patients (OR, 1.042; 95% CI, 1.025–1.060; *P* < 0.001), other independent risk factors included PaO_2_/FiO_2_, Hb, lactate, and use of vasoactive drugs (all *P* < 0.05). Moreover, we found that the duration of IMV was longer in patients with high NLR (8[IQR, 3–13], 10[IQR, 6–16], respectively, *P*=0.025).

**Conclusions:**

Our results revealed that high baseline NLR level was significantly correlated with an increased risk of IMV in patients with ARDS. Furthermore, higher NLR was associated with prolonged duration of IMV in patients with ARDS.

## 1. Introduction

Acute respiratory distress syndrome (ARDS) is a sudden and fatal illness that makes it difficult to get enough oxygen [1], with a mortality rate ranging from 35% to 40% [2, 3]. ARDS severity is classified by the oxygenation index (PaO_2_/FiO_2_), as severe (PaO_2_/FiO_2_ ratio ≤100 mmHg), moderate (PaO_2_/FiO_2_ ratio of 101–200 mmHg), and mild (PaO_2_/FiO_2_ ratio of 201–300 mmHg), with mortality being increased with greater ARDS severity [4, 5]. Most ARDS patients require noninvasive ventilation (NIV) or invasive mechanical ventilation (IMV) to decrease work of breathing and restore gas exchange [6]. Ventilator management differed among the ARDS severity groups. NIV can be implemented outside the intensive care units (ICU) and appear to be effective and safe in mild-to-moderate hypoxemia (PaO_2_/FiO_2_ >150 mmHg), but they can yield delayed intubation with increased mortality in a significant proportion of moderate-to-severe hypoxemia (PaO_2_/FiO_2_ ≤150 mmHg). IMV is commonly used in moderate-to-severe individuals [7]. However, patients receiving IMV often have an increased length of hospital stay and mortality [8]. Therefore, strict physiological monitoring and appropriate ventilation strategies based on patients' individual situations are extremely necessary.

The neutrophil-to-lymphocyte ratio (NLR) is a novel marker of systemic inflammatory response and is defined as the absolute neutrophil count divided by the absolute lymphocyte count, with normal NLR values ranging from 0.78 to 3.53 in an adult, nongeriatric population in good health [9]. Increased NLR has been proven to be valuable in the prognosis of several types of cancers [10], inflammatory diseases [11], and cardiovascular diseases [12]. In addition, an interim analysis of an ongoing prospective study showed that NLR at admission can predict the early requirement of IMV in patients with coronavirus disease 2019 (COVID-19) [13]. To our knowledge, the relationship between NLR and ventilatory support in ARDS patients has not yet been evaluated. Therefore, the purpose of the present study was to assess the performance of baseline NLR for ARDS patients and reveal the relationship between NLR level and the requirement and duration of IMV during hospitalization.

## 2. Methods

### 2.1. Study Design and Patient Population

**Figure 1:**
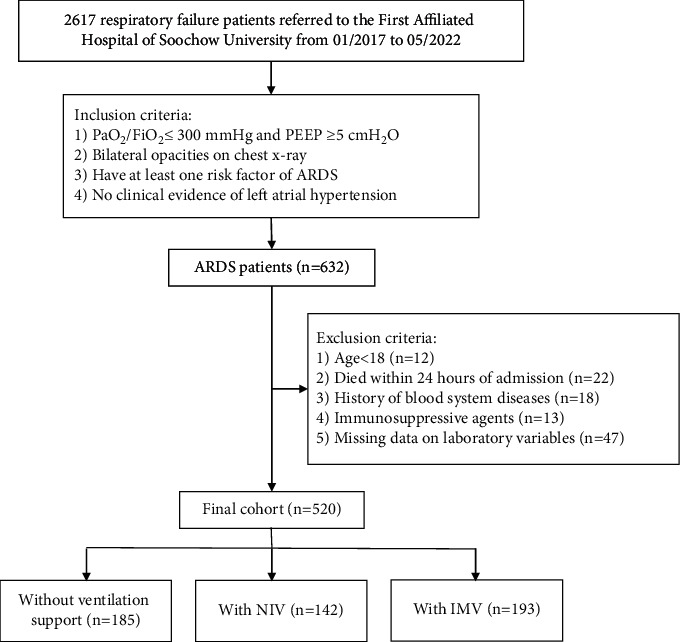
Research flowchart. ARDS, acute respiratory distress syndrome; NIV, noninvasive ventilation; IMV, invasive mechanical ventilation.

This retrospective cohort study was approved by the Clinical Research Ethics Committee of First Affiliated Hospital of Soochow University (Jiangsu, China). Between January 2017 and May 2022, 2617 patients were diagnosed with respiratory failure in the First Affiliated Hospital of Soochow University. The medical records of all patients were reviewed by two attending physicians in the Department of Critical Care Medicine and then 632 patients fulfilled the inclusion criteria based on the Berlin definition [5] of ARDS (new or worsening respiratory symptoms began within 1 week of a known clinical insult, bilateral opacities on chest X-ray, PaO_2_/FiO_2_ less than 300 mmHg with positive end-expiratory pressure being more than 5 cmH_2_O, no clinical evidence of left atrial hypertension, and no other explanation for these findings). We excluded individuals who were with history of blood system diseases, were less than 18 years old, died within 24 h of admission, received immunosuppressant treatments, and were with incomplete medical records. Ultimately, 112 patients were excluded and 520 patients with ARDS (193 patients with IMV, which included 26 patients who required IMV after a failed trial of NIV; 142 patients with NIV; 185 patients without ventilatory support) were enrolled in our study (Figure 1).

### 2.2. Data Extraction

Demographic characteristics (age, gender), past medical history, the risk factors of ARDS, types of infection, sequential organ failure assessment (SOFA) score, acute physiology and chronic health evaluation (APACHE) II score, clinical interventions, and laboratory test results (including PaO_2_/FiO_2_, white blood cell counts, neutrophil counts, monocyte counts, lymphocyte counts, hemoglobin, red cell distribution width, hematocrit, platelets, lactate, albumin, aspartate aminotransferase, alanine aminotransferase, creatinine, and blood urea nitrogen) for participants were collected. All parameters were first measured within 24 hours after the ARDS diagnosis, and all patients were followed up for 28 days. Ventilation strategies (tracheostomy, endotracheal intubation, noninvasive positive pressure ventilation, and high-flow nasal cannula) used during hospitalization and clinical outcomes (duration of ventilation, hospital length of stay, and 28-day mortality) were recorded retrospectively. The indications for mechanical ventilation [14, 15] after diagnosis of ARDS were condition that continued to deteriorate despite aggressive treatments, abnormal respiration, and arterial oxygen tension (Pa0_2_) less than 50 mmHg. Indications may vary according to the acuity of onset of respiratory failure and the underlying disease pathology. MLR and NLR were calculated. Steroid therapy was defined as at least a dose (≥0.5 mg/kg) of methylprednisolone [16, 17]. Vasoactive drugs included epinephrine, norepinephrine, vasopressin, dobutamine, dopamine, and phenylephrine.

### 2.3. Statistical Analysis

SPSS software version 24.0 (SPSS Inc, Chicago, IL) and MedCalc software (version 19.0.4) were used for processing data. Differences in continuous variables were analysed using Student's *t*-test or Mann–Whitney *U* test based on variable distribution, presented as mean ± standard deviation or medians (quartiles). Meanwhile, the differences between categorical variables were compared using Pearson *x*^2^ test or Fisher's exact method and described as frequency and percentage. Univariate and multivariate logistic regression analysis were generated to identify the independent influence factors for requirement of IMV. Multivariate logistic regression analysis used the forward logistic regression method (entering a variable if *P* values are less than 0.05, removing a variable if *P* values are more than 0.10). Binary logistic regression analysis was used to combine covariates. In addition, prognostic values of baseline model and the combination of baseline model and NLR were calculated by operating characteristic curve (ROC) and generated area under the curve (AUC) for different models then compared them using DeLong's test. Values of *P* < 0.05 were considered statistically significant. STROBE checklist for observational studies was used for this manuscript (Table S1).

## 3. Results

### 3.1. Baseline Characteristics of Patients

A total of 520 ARDS patients were included in our study and were divided into three groups according to the ventilation strategies used during hospitalization (Figure 1). The baseline characteristics of the non-MV and IMV groups are displayed in Table 1. Compared with patients without MV, patients with IMV were more likely to be male (*P*=0.012), had lower PaO_2_/FiO_2_ ratio, higher NLR values, and higher SOFA scores (both *P* < 0.001). Laboratory parameters (hemoglobin, hematocrit, platelets, lactate, and blood urea nitrogen) had significant statistical differences among different groups. Within the first 24 h after ARDS diagnosis, the IMV group was more likely to use steroid (59.6% vs. 49.2%; *P*=0.042) and vasoactive drugs (45.1% vs. 14.6%; *P* < 0.001). Furthermore, categories of ARDS, 28-day mortality, and hospital length of stay in the two groups were significantly different (*P* < 0.001, *P*=0.001, *P*=0.002, respectively).

The characteristics of the NIV and IMV groups are summarized in Table 2. Patients in the IMV group had higher NLR values (*P*=0.001), lower PaO_2_/FiO_2_ ratio (126, IQR: 82.94–184.62 vs 139, IQR:100–193.68, *P*=0.040), higher severity of ARDS, and higher SOFA scores (8, IQR: 6–10 vs 7, IQR: 6–9, *P*=0.022) and were more likely to use vasoactive drugs (45.1% vs. 28.2%; *P*=0.002) within 24 h after the ARDS diagnosis compared with NIV group. Furthermore, patients with IMV had higher 28-day mortality and longer hospital length of stay (*P*=0.023, *P*=0.013, respectively). Details about baseline characteristics of the non-IMV and IMV groups are shown in Supplementary Material Table S2.

### 3.2. Relationship between Baseline NLR and High Risk of IMV

Logistic regression analysis was carried out to screen the independent risk factors for requirement of IMV in ARDS patients. Covariates with *P* value less than 0.1 in univariate logistic regression analysis were included in the multivariate logistic regression analysis. In the multivariate logistic regression model, the PaO_2_/FiO_2_ (OR, 0.993; 95%CI, 0.989–0.996; *P* < 0.001), the Hb (OR, 0.989; 95%CI, 0.981–0.998; *P*=0.012), the NLR (OR, 1.042; 95%CI, 1.025–1.060; *P* < 0.001), the lactate (OR, 1.160; 95%CI, 1.063–1.265; *P*=0.001), and the vasoactive drugs (OR, 2.504; 95% CI, 1.601–3.917; *P* < 0.001) were covariates statistically associated with the requirement of IMV in ARDS patients (Table S3, Table 3).

### 3.3. Incremental Prognostic Value of NLR

**Figure 2:**
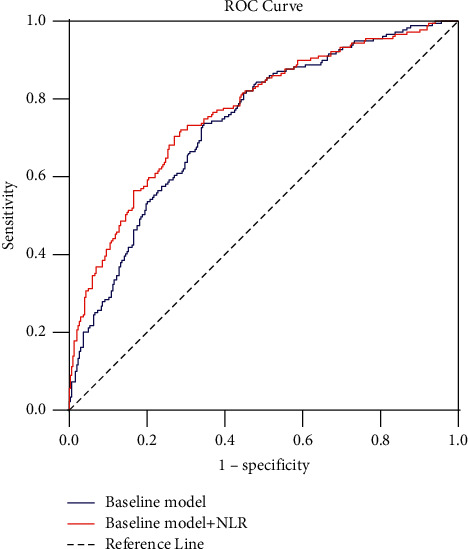
ROC curves for the baseline model and baseline model + NLR for predicting the requirement of invasive mechanical ventilation in patients with ARDS. ROC, receiver operating characteristics; NLR, neutrophil-to-lymphocyte ratio; baseline model + NLR: the integration parameters of baseline model and NLR; ARDS, acute respiratory distress syndrome.

We used receiver operating characteristic (ROC) curves to explore the incremental prognostic value of NLR for ARDS patients (Figure 2). The baseline model included variables that were significant in multivariate logistic regression analysis, such as PaO_2_/FiO_2_, Hb, lactate, and vasoactive drugs. The area under the curve (AUC) was calculated as 0.738 (95% CI: 0.696–0.777; *P* < 0.001) in the baseline model. With NLR in combination with baseline model, the AUC was calculated as 0.768 (95% CI: 0.728–0.805; *P* < 0.001). Compared with baseline model, the inclusion of NLR resulted in a significant increase in the AUC (*P*=0.0097, Table 4).

### 3.4. Baseline Characteristics of IMV Group According to the Different Level of NLR

We divided all the IMV group (*n* = 193) into two subgroups according to the median value of NLR (=14.95), including low NLR group (NLR ≤14.95, *n* = 97) and high NLR group (NLR >14.95, *n* = 96). As shown in Table 5, the duration of IMV was longer in patients with high NLR compared with low NLR group (10[IQR, 6–16], 8[IQR, 3–13], respectively, *P*=0.025). Patients with high NLR had lower PaO_2_/FiO_2_ ratio and lower Alb levels (P = 0.024, *P* < 0.001, respectively). Furthermore, MLR, NLR, age, and 28-day mortality were significantly elevated in high NLR patients compared with low NLR patients. Besides, categories of ARDS in the two subgroups were significantly different (*P*=0.005).

## 4. Discussion

In the current study, we demonstrated that the baseline NLR after diagnosis of ARDS was independently associated with an increased risk of IMV; other independent risk factors included PaO_2_/FiO_2_ ratio, Hb, lactate, and the use of vasoactive drugs. Additionally, higher NLR was associated with prolonged duration of IMV in patients with ARDS.

ARDS is a sudden and fatal disease with an increasing incidence rate, ranging from 20% to 40% and associated with high mortality (ranging from 35% to 40%) [2, 3], as dramatically highlighted by the ongoing COVID-19 pandemic [18, 19]. The histologic characteristic of ARDS is widely considered to be “diffuse alveolar damage,” which is initially driven by dysregulated inflammation response [20]. In the acute stage of ARDS, tiny blood vessels in the lung become leaky, causing protein-rich fluid to fill up the smallest air sacs in the lung (called alveoli), and this is followed by interstitial widening by oedema and then fibroblast infiltration in the subacute phase [21]. COVID-19-related ARDS also causes the typical ARDS pathological changes of diffuse alveolar damage in the lung [22, 23]. As a result, the lung cannot effectively provide oxygen to the rest of the body and clear carbon dioxide, which means that many ARDS patients require ventilator support with NIV or IMV to help them breathe [1]. A study based on the Spanish National Hospital Discharge Database investigated the epidemiological trends in MV use in Spain from 2001 to 2015, which showed an increase in the utilization of NIV while the use of IMV has decreased, and patients who received IMV had a higher in-hospital mortality than those who received NIV [24]. In ARDS, NIV has been shown to be capable of preventing endotracheal intubation in patients with mild-to-moderate hypoxemia (PaO_2_/FiO_2_ >150 mmHg), which is effective and safe. However, for patients with moderate-to-severe hypoxemia (PaO_2_/FiO_2_ ≤150 mmHg), the role of NIV strategies remains unclear [7]. Clinical outcome improves when NIV successfully allows avoiding endotracheal intubation. However, if IMV is needed after a failing trial of NIV, mortality is increased [25]. Consequently, it is important for clinicians to tailor interventions based on patients' individual requirements that can reduce mortality in patients with ARDS.

NLR is the ratio of neutrophil to lymphocyte count and is a simple combined index that can be easily used to evaluate a patient's inflammatory status [26]. A study including 413 active subjects showed that normal NLR value ranges from 0.78 to 3.53 in an adult, nongeriatric population in good health [9]. Furthermore, NLR is a relatively stable parameter that did not significantly change with age and gender [27]. As the result of apoptosis in lymphocytes and an increase in neutrophils, the increase in NLR reflects the balance between lymphocyte count and neutrophil count and may reflect the systemic immune status more comprehensively [28]. Neutrophils are positively associated with the severity of inflammation in ARDS; moreover, they are the first immune cells to be recruited to the site of inflammation after being stimulated by chemokines released from injured lung tissue [23]. Meanwhile, the literature showed that lymphocytes were essential in regulating the appropriate inflammatory response, and low circulating lymphocytes may perpetuate a harmful inflammatory status [29, 30].

Previous studies have shown that increased baseline NLR was associated with poor clinical outcomes in a variety of diseases, such as solid tumors [10, 31], cardiovascular diseases [12], and inflammation-related diseases. Recent studies also reported the relationship between increased NLR and clinical prognosis of ARDS. The conclusions of these studies all suggested that elevated NLR was associated with poor prognosis of ARDS patients. Wang et al. showed that a high NLR (>14) was independently associated with a shorter overall survival in patients with ARDS [32]. Li et al. suggested that NLR was an independent risk factor for predicting 28-day mortality in patients with ARDS [33]. More recently, Zhang et al. found that high NLR (≥14.8) at ICU admission was correlated with higher in-hospital and 30-day mortality in ARDS patients [28]. In addition, a previous study including 81 patients with COVID-19 revealed that NLR >9.8 could predict the overall requirement of IMV [34]. An interim analysis of an ongoing prospective study showed that NLR >4.6 were moderately good in predicting the early requirement of MV within 24 h in COVID-19 patients [13]. Based on the available studies, we supposed that elevated NLR values are associated with IMV in ARDS patients on the evidence of the correlation between NLR-related inflammation and ARDS disease severity.

In the present study, we found the mortality of ARDS patients was quite high, and patients who received IMV had higher 28-day mortality and longer hospital length of stay than those who received NIV or without ventilatory support; this is consistent with previous studies. Besides, patients who received IMV had higher NLR levels, higher severity of ARDS, higher SOFA scores, and lower PaO_2_/FiO_2_ ratios and were more likely to use vasoactive drugs at the time of ARDS diagnosis. Furthermore, there were some significant differences in sex and laboratory parameters (hemoglobin, hematocrit, platelets, lactate, and blood urea nitrogen) compared with non-MV group. We also observed that NLR was a risk factor for requirement of IMV in ARDS independent of Hb, lactate, vasoactive drugs, and Berlin classification. By combining NLR with other independent risk factors, we found it could reach a better predicting effect than using others alone, as indicated by the ROC curve analysis (Figure 2). Severity of ARDS is related to the severity of pulmonary impairment, and more severe ARDS needs a prolonged ventilation time. In our study, patients with high NLR in the IMV group had a higher severity of ARDS and longer duration of IMV. 28-day mortality was also increased compared to those in the low NLR group. The current study suggests that this combined inflammatory-related parameter may be useful for assessing the disease severity, which might be helpful in evaluating the distribution of respiratory equipment in ARDS patients and adjusting clinical treatments and nursing interventions.

There are several limitations in our study. Firstly, this is a retrospective study that included a small number of Chinese patients. Secondly, intubation and initiation of MV care were decided by attending physicians, which may have subjectively impacted on the outcome. Thirdly, NLR was calculated only once at 24 h after the ARDS diagnosis, so whether continuous monitoring NLR level could better predict the requirement and the duration of IMV in patients with ARDS remains unclear. Further studies, particularly prospective studies with greater sample sizes, are needed to reveal the clinical significance of elevated NLR level and the correlation between NLR and IMV in patients with ARDS.

## 5. Conclusions

Based on the results of this study, NLR was elevated in ARDS patients, especially those with IMV. We found that high baseline NLR level was significantly correlated with an increased risk of IMV in patients with ARDS. Furthermore, higher NLR was associated with prolonged duration of IMV. Therefore, this combined inflammatory index can function as a potential parameter to help evaluate the severity of ARDS and predict the requirement and the duration of IMV in patients with ARDS.

## Figures and Tables

**Table 1:** Comparisons of baseline characteristics between the nonmechanical ventilation group and the invasive mechanical ventilation group.

Variables	Non-MV group (*n* = 185)	IMV group (*n* = 193)	*P* value
Age (years)	66 (52–76)	67 (51–75)	0.929
Male, *n* (%)	119 (64.3)	147 (76.2)	0.012
Smoking, *n* (%)	54 (29.2)	48 (24.9)	0.344
Alcohol abuse, *n* (%)	29 (15.7)	39 (20.2)	0.252
Hypertension, *n* (%)	47 (25.4)	57 (29.5)	0.369
Diabetes mellitus, *n* (%)	49 (26.5)	44 (22.8)	0.405
Coronary artery disease, *n* (%)	22 (11.9)	20 (10.4)	0.636
Risk factor, *n* (%)			0.646
Pneumonia	122 (65.9)	130 (67.4)	
Aspiration	7 (3.8)	12 (6.2)	
Sepsis	15 (8.1)	13 (6.7)	
Others	41 (22.2)	38 (19.7)	
Types of infection, *n* (%)			0.665
Bacteria	151 (81.6)	154 (79.8)	
Virus	11 (5.9)	8 (4.1)	
Fungus	6 (3.3)	9 (4.7)	
Unknown	17 (9.2)	22 (11.4)	
PaO_2_/FiO_2_ (mmHg)	200 (160–236.5)	126 (82.94–184.62)	<0.001
Categories of ARDS, *n* (%)			<0.001
Mild	87 (47.0)	36 (18.7)	
Moderate	79 (42.7)	81 (41.9)	
Severe	19 (10.3)	76 (39.4)	
WBC, 10^9^/L	9.22 (6–11.46)	9.8 (7.02–11.84)	0.249
Hb, g/L	118.48 ± 25.49	106.29 ± 25.81	<0.001
RDW, %	13.7 (13–15.1)	13.9 (13.1–15)	0.519
Hematocrit	0.35 ± 0.08	0.33 ± 0.09	0.010
Platelets, 10^9^/L	178.37 (127–261)	161.5 (93–229)	0.013
Lactate, mmol/L	2 (1.25–3.37)	2.54 (1.4–4.46)	0.017
Alb, g/L	31.58 ± 6.25	30.35 ± 7.02	0.073
MLR	0.51 (0.32–0.80)	0.59 (0.30–0.93)	0.147
NLR	10.36 (4.99–15.88)	14.95 (7.73–27.99)	<0.001
AST	39.45 (21.25–107.53)	47.6 (24.68–107.75)	0.204
ALT	38.45 (20–103.38)	45.7 (20.08–107.4)	0.566
Cr, *μ*mol/L	103.07 (54.9–183)	84.5 (56.35–174.25)	0.962
BUN, mmol/L	9.1 (5.36–15.73)	11 (7–17.05)	0.011
APACHE II score	12 (8–15)	13 (9–17)	0.078
SOFA score	7 (5–8)	8 (6–10)	<0.001
Intervention (1^st^24 h), *n* (%)			
Steroid^a^	91 (49.2)	115 (59.6)	0.042
Alimentotherapy	92 (49.7)	112 (58.0)	0.106
Vasoactive drugs^b^	27 (14.6)	87 (45.1)	<0.001
CRRT	20 (10.8)	34 (17.6)	0.059
Outcomes			
28-day mortality, *n* (%)	40 (21.6)	73 (37.8)	0.001
Hospital length of stay	14 (9–20)	18 (11–26)	0.002

^a^Steroid therapy was defined as at least a dose (≥0.5 mg/kg) of methylprednisolone. ^b^Vasoactive drugs include epinephrine, norepinephrine, vasopressin, dobutamine, dopamine, and phenylephrine. MV, mechanical ventilation; IMV, invasive mechanical ventilation; WBC, white blood cell; Hb, hemoglobin; RDW, red cell distribution width; Alb, albumin; MLR, monocyte-to-lymphocyte ratio; NLR, neutrophil-to-lymphocyte ratio; AST, aspartate aminotransferase; ALT, alanine aminotransferase; Cr, creatinine; BUN, blood urea nitrogen; APACHE II, acute physiology and chronic health evaluation II; SOFA, sequential organ failure assessment; CRRT, continuous renal replacement therapy.

**Table 2:** Comparisons of baseline characteristics between the noninvasive ventilation group and the invasive mechanical ventilation group.

Variables	NIV group (*n* = 142)	IMV group (*n* = 193)	*P* value
Age (years)	68 (60–77)	67 (51–75)	0.134
Male, *n* (%)	97 (68.3)	147 (76.2)	0.110
Smoking, *n* (%)	39 (27.5)	48 (24.9)	0.593
Alcohol abuse, *n* (%)	31 (21.8)	39 (20.2)	0.718
Hypertension, *n* (%)	39 (27.5)	57 (29.5)	0.679
Diabetes mellitus, *n* (%)	34 (23.9)	44 (22.8)	0.806
Coronary artery disease, *n* (%)	17 (12.0)	20 (10.4)	0.642
Risk factor, *n* (%)			0.008
Pneumonia	118 (83.1)	130 (67.4)	
Aspiration	4 (2.8)	12 (6.2)	
Sepsis	8 (5.6)	13 (6.7)	
Others	12 (8.5)	38 (19.7)	
Types of infection, *n* (%)			0.795
Bacteria	115 (80.9)	154 (79.8)	
Virus	4 (2.9)	8 (4.1)	
Fungus	9 (6.3)	9 (4.7)	
Unknown	14 (9.9)	22 (11.4)	
PaO_2_/FiO_2_ (mmHg)	139 (100–193.68)	126 (82.94–184.62)	0.040
Categories of ARDS, *n* (%)			0.036
Mild	30 (21.1)	36 (18.7)	
Moderate	75 (52.8)	81 (41.9)	
Severe	37 (26.1)	76 (39.4)	
WBC, 10^9^/L	9.62 (7.73–11.44)	9.8 (7.02–11.84)	0.549
Hb, g/L	106.58 ± 23.78	106.29 ± 25.81	0.919
RDW, %	13.8 (12.9–15.3)	13.9 (13.1–15)	0.786
Hematocrit	0.33 ± 0.08	0.33 ± 0.09	0.535
Platelets, 10^9^/L	155.5 (123.12–228)	161.5 (93–229)	0.413
Lactate, mmol/L	2.31 (1.4–3.46)	2.54 (1.4–4.46)	0.293
Alb, g/L	32.02 ± 6.42	30.35 ± 7.02	0.026
MLR	0.63 (0.33–1.08)	0.59 (0.30–0.93)	0.229
NLR	10.66 (6.56–17.80)	14.95 (7.73–27.99)	0.001
AST	52 (25.3–120)	47.6 (24.68–107.75)	0.525
ALT	34.7 (17.8–84.5)	45.7 (20.08–107.4)	0.220
Cr, *μ*mol/L	105.15 (62.32–186.12)	84.5 (56.35–174.25)	0.423
BUN, mmol/L	11.25 (7.07–17.76)	11 (7–17.05)	0.872
APACHE II score	12 (9–15)	13 (9–17)	0.112
SOFA score	7 (6–9)	8 (6–10)	0.022
Intervention (1^st^24 h), *n* (%)			
Steroid^a^	82 (57.7)	115 (59.6)	0.735
Alimentotherapy	73 (51.4)	112 (58.0)	0.228
Vasoactive drugs^b^	40 (28.2)	87 (45.1)	0.002
CRRT	21 (14.8)	34 (17.6)	0.490
Outcomes			
28-day mortality, *n* (%)	37 (26.1)	73 (37.8)	0.023
Hospital length of stay	14 (9–22)	18 (11–26)	0.013

^a^Steroid therapy was defined as at least a dose (≥0.5 mg/kg) of methylprednisolone. ^b^Vasoactive drugs include epinephrine, norepinephrine, vasopressin, dobutamine, dopamine, and phenylephrine. NIV, noninvasive ventilation; IMV, invasive mechanical ventilation; WBC, white blood cell; Hb, hemoglobin; RDW, red cell distribution width; Alb, albumin; MLR, monocyte-to-lymphocyte ratio; NLR, neutrophil-to-lymphocyte ratio; AST, aspartate aminotransferase; ALT, alanine aminotransferase; Cr, creatinine; BUN, blood urea nitrogen; APACHE II, acute physiology and chronic health evaluation II; SOFA, sequential organ failure assessment; CRRT, continuous renal replacement therapy.

**Table 3:** Influence factors for requirement of IMV in ARDS patients by univariate and multivariate logistic regression analysis.

Variables	Univariate analysis		Multivariate analysis	
	OR (95% CI)	*P* value	OR (95% CI)	*P* value
Male, *n* (%)	1.642 (1.098–2.456)	0.016		
PaO_2_/FiO_2_ (mmHg)	0.991 (0.988–0.994)	<0.001	0.993 (0.989–0.996)	<0.001
Hb, g/L	0.989 (0.982–0.996)	0.003	0.989 (0.981–0.998)	0.012
Hematocrit	0.106 (0.012–0.960)	0.046		
Platelets, 10^9^/L	0.998 (0.996–1.000)	0.061		
Lactate, mmol/L	1.166 (1.075–1.263)	<0.001	1.160 (1.063–1.265)	0.001
Alb, g/L	0.968 (0.942–0.994)	0.018		
NLR	1.046 (1.031–1.062)	<0.001	1.042 (1.025–1.060)	<0.001
APACHE II score	1.043 (1.009–1.078)	0.012		
SOFA score	1.137 (1.073–1.205)	<0.001		
Alimentotherapy	1.358 (0.948–1.943)	0.095		
Vasoactive drugs^b^	3.185 (2.155–4.707)	<0.001	2.504 (1.601–3.917)	<0.001

^b^Vasoactive drugs include epinephrine, norepinephrine, vasopressin, dobutamine, dopamine, and phenylephrine. IMV, invasive mechanical ventilation; ARDS, acute respiratory distress syndrome; Hb, hemoglobin; Alb, albumin; NLR, neutrophil-to-lymphocyte ratio; APACHE II, acute physiology and chronic health evaluation II; SOFA, sequential organ failure assessment; OR, odds ratio; CI, confidence interval.

**Table 4:** The value of indicators in predicting the requirement of IMV in patients with ARDS.

Parameters	AUC	95%CI	*P* value
Baseline model^a^	0.738	0.696–0.777	RF
+NLR	0.768	0.728–0.805	0.0097

^a^The baseline model includes variables that are significant in multivariate logistic regression analysis, including male, PaO_2_/FiO_2_, Hb, lactate, and vasoactive drugs. IMV, invasive mechanical ventilation; ARDS, acute respiratory distress syndrome; NLR, neutrophil-to-lymphocyte ratio; AUC, area under the curve; CI, confidence interval; RF, reference.

**Table 5:** Baseline characteristics of IMV group in different NLR levels.

Variables	Low NLR (NLR ≤14.95, *n* = 97)	High NLR (NLR >14.95, *n* = 96)	*P* value
Age (years)	63 (47–73)	71 (56–77)	0.002
Male, *n* (%)	77 (79.4)	70 (72.9)	0.292
Smoking, *n* (%)	29 (29.9)	19 (19.8)	0.104
Alcohol abuse, *n* (%)	26 (26.8)	13 (13.5)	0.022
Hypertension, *n* (%)	25 (25.8)	32 (33.3)	0.250
Diabetes mellitus, *n* (%)	17 (17.5)	27 (28.1)	0.079
Coronary artery disease, *n* (%)	13 (13.4)	7 (7.3)	0.164
Risk factor, *n* (%)			0.414
Pneumonia	60 (61.9)	70 (72.9)	
Aspiration	7 (7.2)	5 (5.2)	
Sepsis	7 (7.2)	6 (6.3)	
Others	23 (23.7)	15 (15.6)	
Types of infection, *n* (%)			0.432
Bacteria	82 (84.5)	72 (75.0)	
Virus	3 (3.1)	5 (5.2)	
Fungus	3 (3.1)	6 (6.3)	
Unknown	9 (9.3)	13 (13.5)	
PaO_2_/FiO_2_ (mmHg)	140 (89.17–219.84)	115 (81.53–166.83)	0.024
Categories of ARDS, *n* (%)			0.005
Mild	27 (27.8)	9 (9.4)	
Moderate	36 (37.1)	45 (46.9)	
Severe	34 (35.1)	42 (43.8)	
WBC, 10^9^/L	9.69 (5.95–12.13)	9.57 (6.95–11.62)	0.947
Hb, g/L	104.43 ± 27.59	108.18 ± 23.88	0.313
RDW, %	14.05 (13–15.18)	13.7 (13.2–14.8)	0.551
Hematocrit	0.33 ± 0.09	0.33 ± 0.08	0.862
Platelets, 10^9^/L	168.43 ± 105.87	172.20 ± 99.18	0.799
Lactate, mmol/L	2.39 (1.28–4.90)	2.61 (1.5–4.22)	0.864
Alb, g/L	33 (27.5–36.77)	28.4 (23.2–32.4)	<0.001
MLR	0.39 (0.25–0.64)	0.79 (0.48–1.37)	<0.001
NLR	7.79 (3.78–10.80)	27.99 (20.15–47.36)	<0.001
AST	46 (23.4–138)	48 (27.2–86.6)	0.877
ALT	43.6 (20–107.2)	49 (20.4–108)	0.984
Cr, *μ*mol/L	80.3 (52–164.28)	88.85 (60.55–183.91)	0.217
BUN, mmol/L	10.11 (6.71–16.02)	11.83 (6.96–19.28)	0.158
APACHE II score	12 (8–16)	14 (9–18)	0.283
SOFA score	8 (6–10)	8 (6–10)	0.885
Interventions, *n* (%)			
Steroid^a^	57 (58.8)	58 (60.4)	0.815
Alimentotherapy	52 (53.6)	60 (62.5)	0.211
Vasoactive drugs^b^	41 (42.3)	46 (47.9)	0.430
CRRT	16 (16.5)	18 (18.8)	0.681
28-day mortality, *n* (%)	29 (29.9)	44 (45.8)	0.022
Duration of ventilation	8 (3–13)	10 (6–16)	0.025
Hospital length of stay	16 (8–25)	18 (12–26)	0.173

^a^Steroid therapy was defined as at least a dose (≥0.5 mg/kg) of methylprednisolone. ^b^Vasoactive drugs include epinephrine, norepinephrine, vasopressin, dobutamine, dopamine, and phenylephrine. IMV, invasive mechanical ventilation; ARDS, acute respiratory distress syndrome; WBC, white blood cell; Hb, hemoglobin; RDW, red cell distribution width; Alb, albumin; MLR, monocyte-to-lymphocyte ratio; NLR, neutrophil-to-lymphocyte ratio; AST, aspartate aminotransferase; ALT, alanine aminotransferase; Cr, creatinine; BUN, blood urea nitrogen; APACHE II, acute physiology and chronic health evaluation II; SOFA, sequential organ failure assessment; CRRT, continuous renal replacement therapy.

## Data Availability

The datasets used and/or analysed during the current study are available from the corresponding author upon reasonable request.

## Supplementary Materials

Supplementary Materials Table S1: STROBE Statement: checklist of items that should be included in reports of cohort studies. Table S2: clinical and demographic characteristics of the enrolled patients. Table S3: univariate logistic regression analysis of the requirement of IMV in patients with ARDS.

## References

[1] Stevens J. P., Law A., Giannakoulis J. (2018). Acute respiratory distress syndrome. *JAMA*.

[2] Pham T., Rubenfeld G. D. (2017). Fifty years of research in ARDS. The epidemiology of acute respiratory distress syndrome. A 50th birthday review. *American Journal of Respiratory and Critical Care Medicine*.

[3] Sigurdsson M. I., Sigvaldason K., Gunnarsson T. S., Moller A., Sigurdsson G. H. (2013). Acute respiratory distress syndrome: nationwide changes in incidence, treatment and mortality over 23 years. *Acta Anaesthesiologica Scandinavica*.

[4] Bellani G., Laffey J. G., Pham T. (2016). Epidemiology, patterns of care, and mortality for patients with acute respiratory distress syndrome in intensive care units in 50 countries. *JAMA*.

[5] Force A. D. T., Ranieri V. M., Rubenfeld G. D. (2012). Acute respiratory distress syndrome: the Berlin definition. *JAMA*.

[6] Amado-Rodriguez L., Del Busto C., Garcia-Prieto E., Albaiceta G. M. (2017). Mechanical ventilation in acute respiratory distress syndrome: the open lung revisited. *Medicina Intensiva*.

[7] Grieco D. L., Maggiore S. M., Roca O. (2021). Non-invasive ventilatory support and high-flow nasal oxygen as first-line treatment of acute hypoxemic respiratory failure and ARDS. *Intensive Care Medicine*.

[8] Wunsch H., Linde-Zwirble W. T., Angus D. C., Hartman M. E., Milbrandt E. B., Kahn J. M. (2010). The epidemiology of mechanical ventilation use in the United States. *Critical Care Medicine*.

[9] Forget P., Khalifa C., Defour J. P., Latinne D., Van Pel M. C., De Kock M. (2017). What is the normal value of the neutrophil-to-lymphocyte ratio?. *BMC Research Notes*.

[10] Miyamoto R., Inagawa S., Sano N., Tadano S., Adachi S., Yamamoto M. (2018). The neutrophil-to-lymphocyte ratio (NLR) predicts short-term and long-term outcomes in gastric cancer patients. *European Journal of Surgical Oncology*.

[11] Seyit M., Avci E., Nar R. (2021). Neutrophil to lymphocyte ratio, lymphocyte to monocyte ratio and platelet to lymphocyte ratio to predict the severity of COVID-19. *American Journal of Emergency Medicine*.

[12] Haybar H., Pezeshki S. M. S., Saki N. (2019). Evaluation of complete blood count parameters in cardiovascular diseases: an early indicator of prognosis?. *Experimental and Molecular Pathology*.

[13] Wu Z., McGoogan J. M. (2020). Characteristics of and important lessons from the coronavirus disease 2019 (COVID-19) outbreak in China: summary of a report of 72314 cases from the Chinese center for disease control and prevention. *JAMA*.

[14] Barbarash R. A., Smith L. A., Godwin J. E., Sahn S. A. (1990). Mechanical ventilation. *DICP*.

[15] Fan E., Del Sorbo L., Goligher E. C. (2017). An official American thoracic society/European society of intensive care medicine/society of critical care medicine clinical practice guideline: mechanical ventilation in adult patients with acute respiratory distress syndrome. *American Journal of Respiratory and Critical Care Medicine*.

[16] Cao B., Gao H., Zhou B. (2016). Adjuvant corticosteroid treatment in adults with influenza A (H7N9) viral pneumonia. *Critical Care Medicine*.

[17] Tang B. M. P., Craig J. C., Eslick G. D., Seppelt I., McLean A. S. (2009). Use of corticosteroids in acute lung injury and acute respiratory distress syndrome: a systematic review and meta-analysis. *Critical Care Medicine*.

[18] Ferguson N. D., Pham T., Gong M. N. (2020). How severe COVID-19 infection is changing ARDS management. *Intensive Care Medicine*.

[19] Murthy S., Gomersall C. D., Fowler R. A. (2020). Care for critically ill patients with COVID-19. *JAMA*.

[20] Katzenstein A. L., Bloor C. M., Leibow A. A. (1976). Diffuse alveolar damage--the role of oxygen, shock, and related factors. A review. *American Journal Of Pathology*.

[21] Matthay M. A., Zemans R. L. (2011). The acute respiratory distress syndrome: pathogenesis and treatment. *Annual Review of Pathology: Mechanisms of Disease*.

[22] Xu Z., Shi L., Wang Y. (2020). Pathological findings of COVID-19 associated with acute respiratory distress syndrome. *Lancet Respiratory Medicine*.

[23] Yang S. C., Tsai Y. F., Pan Y. L., Hwang T. L. (2021). Understanding the role of neutrophils in acute respiratory distress syndrome. *Biomedical Journal*.

[24] de-Miguel-Diez J., Jimenez-Garcia R., Hernandez-Barrera V. (2020). Trends in mechanical ventilation use and mortality over time in patients receiving mechanical ventilation in Spain from 2001 to 2015. *European Journal of Internal Medicine*.

[25] Rochwerg B., Einav S., Chaudhuri D. (2020). The role for high flow nasal cannula as a respiratory support strategy in adults: a clinical practice guideline. *Intensive Care Medicine*.

[26] Forrest E. H. (2017). Letter: there’s something about the neutrophil-to-lymphocyte ratio (NLR). *Alimentary Pharmacology & Therapeutics*.

[27] Luo H., He L., Zhang G. (2019). Normal reference intervals of neutrophil-to-lymphocyte ratio, platelet-to-lymphocyte ratio, lymphocyte-to-monocyte ratio, and systemic immune inflammation index in healthy adults: a large multi-center study from western China. *Clinical Laboratory*.

[28] Zhang W., Wang Y., Li W., Wang G. (2021). The association between the baseline and the change in neutrophil-to-lymphocyte ratio and short-term mortality in patients with acute respiratory distress syndrome. *Frontiers of Medicine*.

[29] Heffernan D. S., Monaghan S. F., Thakkar R. K., Machan J. T., Cioffi W. G., Ayala A. (2012). Failure to normalize lymphopenia following trauma is associated with increased mortality, independent of the leukocytosis pattern. *Critical Care*.

[30] Le Tulzo Y., Pangault C., Gacouin A. (2002). Early circulating lymphocyte apoptosis in human septic shock is associated with poor outcome. *Shock*.

[31] Gulben K., Berberoglu U., Ondes B., Uyar O., Guler O. C., Turanli S. (2020). Preoperative neutrophil-to-lymphocyte ratio as a predictive factor for survival in nonmetastatic colorectal cancer. *Journal of Cancer Research and Therapeutics*.

[32] Wang Y., Ju M., Chen C. (2018). Neutrophil-to-lymphocyte ratio as a prognostic marker in acute respiratory distress syndrome patients: a retrospective study. *Journal of Thoracic Disease*.

[33] Li W., Ai X., Ni Y., Ye Z., Liang Z. (2019). The association between the neutrophil-to-lymphocyte ratio and mortality in patients with acute respiratory distress syndrome: a retrospective cohort study. *Shock*.

[34] Ma A., Cheng J., Yang J., Dong M., Liao X., Kang Y. (2020). Neutrophil-to-lymphocyte ratio as a predictive biomarker for moderate-severe ARDS in severe COVID-19 patients. *Critical Care*.

